# Key genes for modulating information flow play a temporal role as breast tumor coexpression networks are dynamically rewired by letrozole

**DOI:** 10.1186/1755-8794-6-S2-S2

**Published:** 2013-05-07

**Authors:** Nadia M Penrod, Jason H Moore

**Affiliations:** 1 Department of Pharmacology and Toxicology, Geisel School of Medicine at Dartmouth College, HB7937 One Medical Center Dr., Lebanon, NH 03766, USA; 2 Department of Genetics, Geisel School of Medicine at Dartmouth College, HB7937 One Medical Center Dr., Lebanon, NH 03766, USA; 3 Institute for Quantitative Biomedical Sciences, Geisel School of Medicine at Dartmouth College, HB7937 One Medical Center Dr., Lebanon, NH 03766, USA

## Abstract

**Background:**

Genes do not act in isolation but instead as part of complex regulatory networks. To understand how breast tumors adapt to the presence of the drug letrozole, at the molecular level, it is necessary to consider how the expression levels of genes in these networks change relative to one another.

**Methods:**

Using transcriptomic data generated from sequential tumor biopsy samples, taken at diagnosis, following 10-14 days and following 90 days of letrozole treatment, and a pairwise partial correlation statistic, we build temporal gene coexpression networks. We characterize the structure of each network and identify genes that hold prominent positions for maintaining network integrity and controlling information-flow.

**Results:**

Letrozole treatment leads to extensive rewiring of the breast tumor coexpression network. Approximately 20% of gene-gene relationships are conserved over time in the presence of letrozole while 80% of relationships are condition dependent. The positions of influence within the networks are transiently held with few genes stably maintaining high centrality scores across the three time points.

**Conclusions:**

Genes integral for maintaining network integrity and controlling information flow are dynamically changing as the breast tumor coexpression network adapts to perturbation by the drug letrozole.

## Background

Gene signatures are routinely used to predict drug action, patient relapse, overall survival, and treatment response to stratify breast cancer patients for tailored therapies [1]. Gene signatures are derived from genome-wide expression profiles that capture the global state of gene transcription at a given moment in time. The utility of these genome-wide measures largely depends on the computational methods used to transform the data into an interpretable form. Conventional analysis methods detect patterns of genes whose differential expression can distinguish between various biological conditions, for example, drug treated and untreated samples. Notably, prognostic or predictive gene signatures with comparable accuracies tend to have few, if any, genes in common [2]. However, gene expression levels are highly correlated and these signatures do often recapitulate the same signaling pathways reflecting that genes are not acting in isolation but as components of larger gene regulatory networks.

It is well established that biological function arises from context-dependent interactions among the component parts of the cell [3]. The snapshots of global gene expression provided by microarray data are a source from which these context-dependent interactions can be identified. Genes that are being coregulated will have correlated expression values and a tendency to function as part of the same or related regulatory processes [4]. By focusing on these coexpression relationships in breast cancer we can maximize the amount of information gained from genomic data.

An estimated two-thirds of breast cancers are estrogen receptor positive (ER^+^) enabling them to respond to mitogenic estrogen signaling. Estrogen regulates cell growth and differentiation influencing the development and progression of breast cancer by binding to and activating ERs. ERs regulate gene expression through the activation or repression of gene transcription and participate in cell signaling processes [5]. Anti-estrogen therapies that block the synthesis of estrogen, such as letrozole, are routinely used to treat breast cancers.

Letrozole is a third-generation non-steroidal aromatase inhibitor. Through competitive, reversible binding to the aromatase enzyme letrozole blocks the production of estrogen by inhibiting the conversion of androgens into estrogens. While it is possible to reduce the volume of ER^+ ^tumors by inhibiting the production of estrogen it is important to consider that estrogen plays an integral role in the normal physiology of women by controlling diverse processes such as cholesterol production and maintenance of bone density [6]. This suggests that the inhibition of estrogen will lead to global gene expression changes in the affected tissues including, but not limited to, those that block tumor growth.

A study carried out by the Breast Research Group at the University of Edinburgh has produced gene expression profiles of ER^+ ^tumors from postmenopausal women in the course of neoadjuvant treatment with the aromatase inhibitor letrozole [7]. RNA was isolated from sequential tumor biopsies taken before treatment and after 10-14 days and 90 days of treatment and used for microarray analysis. Sets of differentially expressed genes, based on frequency, magnitude, and significance, were collated with respect to time. When classified by the Gene Ontology (GO) database it was shown that these gene sets contain representatives of diverse biological pathways.

In prior work, Miller et al. found that markers of estrogen sensitivity were largely downregulated by letrozole regardless of whether a tumor responded to the drug [8]. Notably, no single gene was able to consistently discriminate between clinically responsive and resistant tumor samples [9]. In addition, a significant reduction in markers of proliferation by letrozole did not correlate with clinical response [10]. These findings are consistent with the larger BIG 1-98 trial which concluded that letrozole induced changes in ER, progesterone receptor (PGR) and Ki-67 expression levels do not correlate with clinical response [11]. Taken together these data illustrate the complexity of estrogen responsive gene regulatory networks and suggest that there is a letrozole induced gene signature regardless of response status.

By focusing on gene coexpression relationships as a complement to differential expression we are able to reveal a global picture of how the network of affected genes responds to letrozole perturbation. In this study we determine how the expression of genes change relative to one another in response to the aromatase inhibitor letrozole. We look at how the gene coexpression networks from ER^+ ^breast tumor samples are rewired in response to letrozole treatment over time. And we determine how this rewiring changes the set of genes impacting information flow across the coexpression networks.

## Methods

### Data description

Publicly available gene expression data were used for differential expression and coexpression analyses. The transcriptomic data were generated from sequential biopsies of ER^+ ^breast tumors from postmenopausal women during neoadjuvant treatment with letrozole [7,8]. Core biopsy samples were collected at diagnosis and following 10-14 days and 90 days of treatment with 2.5 mg/day of letrozole. A total of 176 samples were available including 58 samples collected at diagnosis (pre-treatment), 58 samples collected on treatment day 14 (mid-treatment), and 60 samples collected on treatment day 90 (post-treatment). RNA was extracted from biopsies containing at least 20% malignant tissue then amplified and hybridized to Affymetrix HG-U133A GeneChip arrays. These data capture temporal gene expression profiles as they change in response to the presence of letrozole within a natural physiological context.

### Data processing

We downloaded the raw CEL files from the Gene Expression Omnibus website (GEO GSE20181). We use a custom CDF from Dai et al. to get the most recent probe annotations and to ensure a a unique mapping between probes and genes [12]. The R implementation of RMA is used to background correct, normalize, and summarize probe set data [13]. We filter the processed data to remove Affymetrix spike-in controls.

### Differential expression analysis

To identify a set of genes that are differentially expressed by the drug letrozole, we compare the processed expression levels of each gene between the pre-treatment samples and the post-treatment (90 days on drug) samples by linear modeling. This method was chosen based on it's performance across a variety of sample sizes and noise levels [14]. We set an FDR threshold of 5% to correct for multiple hypothesis testing. These analyses are performed in R with the Limma package [13,15].

To identify GO biological processes that are overrepresented in the up-regulated and down-regulated gene lists we use the Database for Annotation, Visualization and Integrated Discovery (DAVID) [16]. Reported p-values reflect the EASE score (a modified Fisher Exact p-value) provided by DAVID.

### Coexpression by partial correlation

To identify direct gene-gene coexpression relationships among the set of differentially expressed genes over the course of letrozole treatment we calculate pairwise 1st-order Spearman partial correlation coefficients using the expression levels of these genes across patient samples at each of the three time points separately [17]. This allows us to generate an independent set of coexpression relationships for pre-treatment, mid-treatment, and post-treatment samples. To balance Type I and Type II error we set a significance threshold of *α *= 0.01 based on simulations carried out by de la Fuente et al. [17]. To validate this threshold we use permutation testing. Permutation tests are designed to randomize the expression values for each gene, across samples, within each time point. Following randomization we calculate all pairwise 1st-order Spearman partial correlation coefficients and count the number that meet our significance threshold. This process is repeated 1000 times to generate a null distribution. The observed number of significant pairwise partial correlation relationships, for each time point, fall outside the upper bound of the matched null distribution.

### Coexpression networks

We build three coexpression networks, one for each time point, where each node represents one of the differentially expressed genes and each undirected link indicates a significant direct correlation between the expression levels of the pair of genes it connects. We define hubs as nodes that are statistical outliers by degree and bottlenecks as nodes that are statistical outliers by betweenness centrality (Figure 2). We subcategorize nodes into hub bottlenecks, or nodes that are statistical outliers in both degree and betweenness centralities, hub nonbottlenecks, or nodes that are outliers in degree but not betweeness centrality and nonhub bottlenecks, or nodes that are statistical outliers by betweenness centrality but not degree. All statistical analyses are performed in R and network characterization is performed with the igraph package [18,19].

## Results and discussion

We begin by identifying the subset of genes whose transcript levels change in the presence of the drug letrozole. Using linear modeling we find that 1044 genes are differentially expressed following 90 days of letrozole treatment in these tumors. This gene set is comprised of 575 upregulated genes and 469 downregulated genes. Biological process annotation through the Gene Ontology shows the upregulated genes are enriched for cell migration (*p *= 2.2E-7), positive regulation of gene transcription (*p *= 1.3E-6), polysaccharide binding (*p *= 1.0E-6), cell morphogenesis involved in differentiation (*p *= 2.9E-5), ovulation cycle (*p *= 1.8E-3) and blood coagulation (*p *= 2.4E-3). The downregulated genes are enriched for mitosis (*p *= 5.9E-28), micro-tubule cytoskeleton organization (*p *= 1.2E-15), DNA repair (*p *= 1.9E-7), protein targeting to the mitochondria (*p *= 4.2E-5), nucleotide biosynthesis process (*p *= 9.4E-5) and nucleosome assembly (*p *= 1.4E-4). These biological processes are consistent with the broad roles estrogen plays in the regulation of gene transcription and within cell signaling pathways. It is also indicative of the complexity of estrogen sensitive gene networks. We can study these networks by modeling gene-gene relationships over time as the system is perturbed by letrozole. By focusing on the genes that are differentially expressed in the presence of letrozole we can capture dynamic relationships.

First, we catalog gene coexpression relationships among the set of differentially expressed genes at each of the three time points (i.e. prior to letrozole treatment (pre-treatment), following 10-14 days (mid-treatment) or following 90 days (post-treatment) of letrozole treatment). Because we know relationships between genes are complex, we cannot assume the correlation relationships between gene pairs will be linear. Therefore, we use the nonparametric Spearman's rank correlation to determine coexpression. In addition to capturing nonlinear relationships, this method also avoids some of the bias introduced by experimental design and data preprocessing methods that have been shown to affect Pearson's correlation coefficient [20]. We couple the rank correlation with the 1st-order partial correlation to remove the effects of common regulators and to ensure we are only including direct gene-gene relationships [17].

Second, we use these relationships to assemble three coexpression networks, one for each time point. The nodes of the networks represent genes and the links indicate direct correlations between the expression levels of the pairs of genes they connect. We calculate coexpression between all pairs of the 1044 differentially expressed genes which means each network has 1044 nodes and can have up to 544,446 links. However, only those coexpression relationships that reach statistical significance are included as links within a given network. This results in three networks that have the same set of nodes but varying numbers of links.

These networks map all detectable coexpression relationships occurring at each of three sequential time points over the course of letrozole treatment enabling us to track how the network is rewired as it adapts to the presence of the drug. Furthermore, we can use structural properties of the network to prioritize genes for further analysis that are likely to play functionally relevant roles in maintaining network integrity and controlling information flow throughout the system.

**Figure 1:**
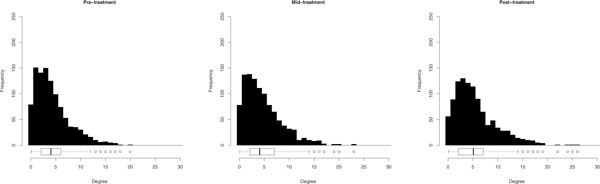
**Degree distributions**. Changes in the degree distributions of breast tumor coexpression networks during the course of neoadjuvant treatment with letrozole. Coexpression networks are built from gene expression data measured at diagnosis (Pre-treatment), following 10-14 days of letrozole treatment (Mid-treatment), and following 90 days of letrozole treatment (Post-treatment). Each network contains 1044 nodes representing the set of genes differentially expressed following 90 days of letrozole treatment. The number of links vary over the course of letrozole treatment with 2262, 2242, 2858 links in the pre-treatment, mid-treatment and post-treatment graphs, respectively.

The pre-treatment, mid-treatment, and post-treatment networks have 2262, 2462, 2858 links, respectively. In each network the distribution of links among the 1044 nodes is not uniform but instead exhibits a heavy-tail, an indication that the networks have highly connected nodes, or hubs (Figure 1). The degree distributions of the pre-treatment and mid-treatment networks are not signficantly different by the Kolmogorov-Smirnov test (*p *= 0.1356). The degree distribution of the post-treatment network does vary signficantly from the mid-treatment network (*p *= 0.0023). The number of genes with low degree decreases while the average degree increases showing the general trend that more genes tend to have more links in a time-dependent manner in the presence of letrozole (Figure 1).

To determine how the differences in the number of links and connectivities of the networks impact network topology we measure the mean shortest path lengths (i.e. the average shortest distance between all pairs of nodes) and the global clustering coefficients (i.e. the probability that common neighbors of a given node are themselves neighbors).

The mean shortest path length is an estimate of network navigability [21]. We calculate the mean shortest path length within the largest connected component of each network. The size of the largest connected component increases and the mean path length decreases in the presence of letrozole (Table 1). However, the distances between the nodes that are part of the largest connected component are separated on average by approximately five links, regardless of letrozole treatment status. There are statistically significant differences in the distributions of the shortest path lengths between the pre-treatment and mid-treatment networks (p *<*.0001) and between the mid-treatment and post-treatment networks (p *<*.0001). This suggests that the increase in links is not providing alternate routes that shorten the distances between nodes globally but that the path lengths between specific nodes are changing in the presence of letrozole.

**Table 1:** Topological properties

	Number of Nodes	Number of Links	Largest Connected Component	Mean Path Length	Global Clustering Coefficient
Pre-treatment	1044	2262	942	5.16	0.090
Mid-treatment	1044	2462	957	5.12	0.089
Post-treatment	1044	2858	982	4.79	0.103

Characteristics of coexpression networks over the course of treatment with letrozole. Coexpression networks are built from gene expression data measured at diagnosis (Pre-treatment), following 10-14 days of letrozole treatment (Mid-treatment), and following 90 days of letrozole treatment (Post-treatment). The largest connected component is the number of nodes within the largest subgraph. Mean path length is a measure of the average distance between all pairs of nodes within the largest connected component of the network. The global clustering coefficient is the probability that neighboring nodes of a given node are connected.

Each network has a characteristic global clustering coefficient that estimates the probability that adjacent nodes of a given node are connected by a link [21,22]. Biological networks have relatively high clustering coefficients, for example, the neural network of *Caenorhabditis elegans *is close to 30% [23]. In our networks, the global clustering coefficients are low, on the order of 9-10% regardless of letrozole treatment status (Table 1). This indicates that pairs of nodes are gaining or losing links independently as opposed to groups of nodes (i.e. subgraphs) becoming more connected. Even with an increase in the number of links the network is not becoming more cohesive. This suggests that genes are being independently regulated as they react to the presence of letrozole and furthermore, that the relationships of individual genes are the distinguishing characteristics of these networks.

**Figure 2:**
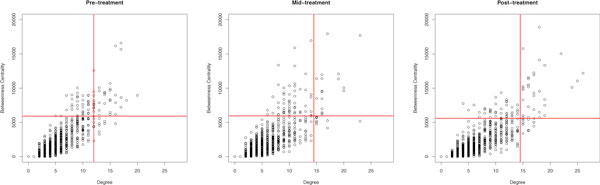
**Centrality distributions**. Genes are categorized based on their degree and betweenness centrality scores. Lines indicate the statistical outlier thresholds for the degree (vertical line) and betweenness centralities (horizontal line). These thresholds are used to categorize genes into hub-bottlenecks (high degree and high betweenness centralities), hub-nonbottlenecks (high degree), nonhub-nonbottlenecks, and nonhub-bottlenecks (high betweenness centrality).

We use bottleneck nodes to survey independent regulation because it has been shown that bottleneck nodes are regulated in a condition-dependent manner in biological networks [24]. We define bottleneck nodes as nodes that are statistical outliers based on their betweenness centrality score (Figure 2). The betweenness centrality score counts the number of shortest paths that cross a given node. The score is an indication of how embedded (i.e. central) a node is within the network.

We also take the hubs of the networks into consideration (Figure 1). As highly connected nodes hubs play an integral role in maintaining network integrity and in mass information transfer. We define hubs as nodes that are statistical outliers in terms of their degree centrality (i.e. number of links).

In protein interaction networks hubs and bottlenecks have both been associated with functional essentiality [24-26]. Here we are also interested in their ability to influence the propagation of signals across a network. Because hubs have many neighbors, signals that reach them can be quickly propagated to a large number of nearby nodes. Bottlenecks may or may not have many neighbors but they are positioned to create efficient paths of information-flow throughout an entire network. In addition, we make the distinction between hub bottlenecks (i.e. high degree and betweenness centralities), hub nonbottlenecks (i.e. high degree) and nonhub bottlenecks (i.e. high betweenness) (Figure 2).

**Figure 3:**
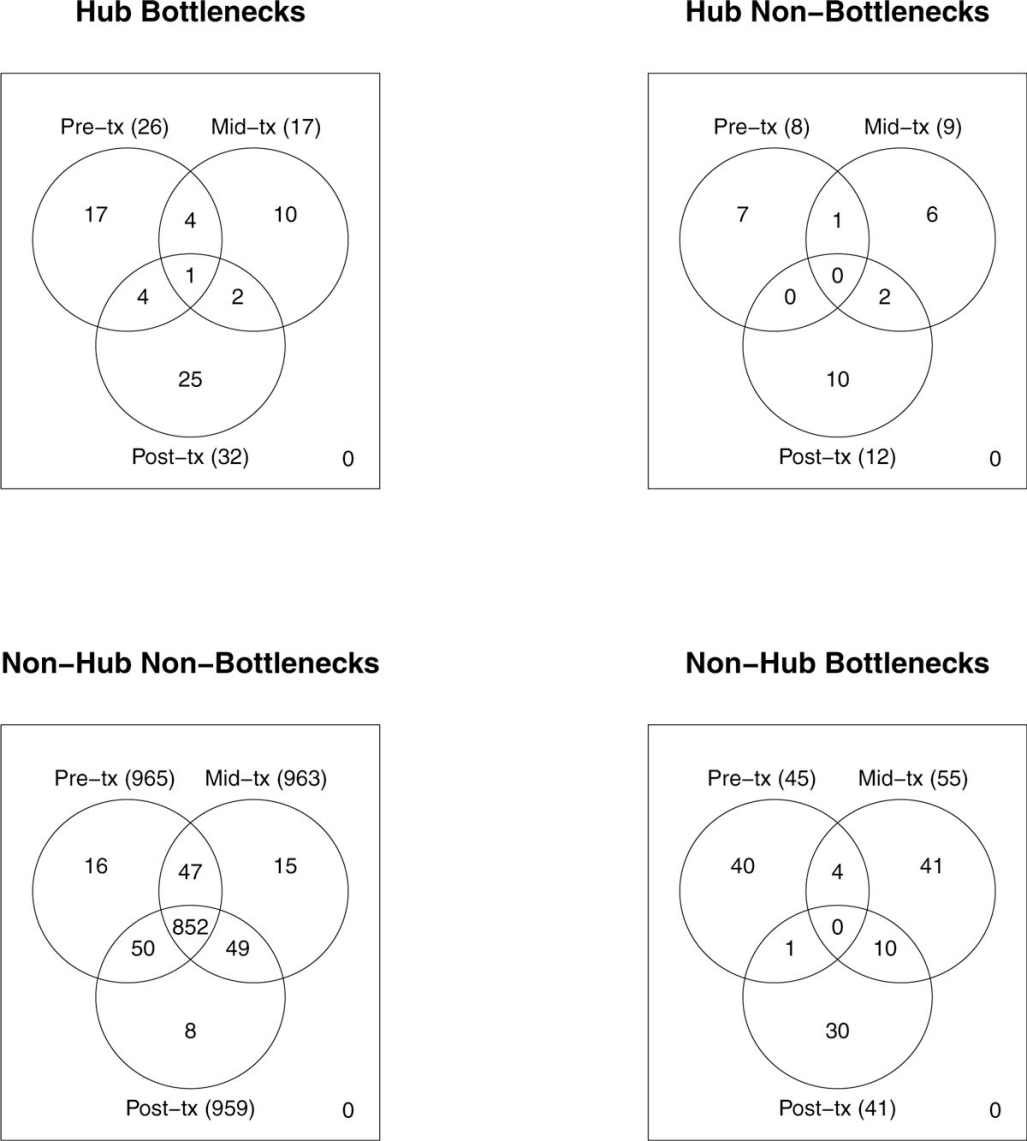
**Common central genes**. Venn diagrams illustrating the number of genes common to each centrality category between time points.

The majority of hubs and bottlenecks only hold these prominent positions at one of the three time points (Figure 3). Accordingly, key genes that are maintaining network integrity and modulating information-flow change over time in the presence of the drug.

**Figure 4:**
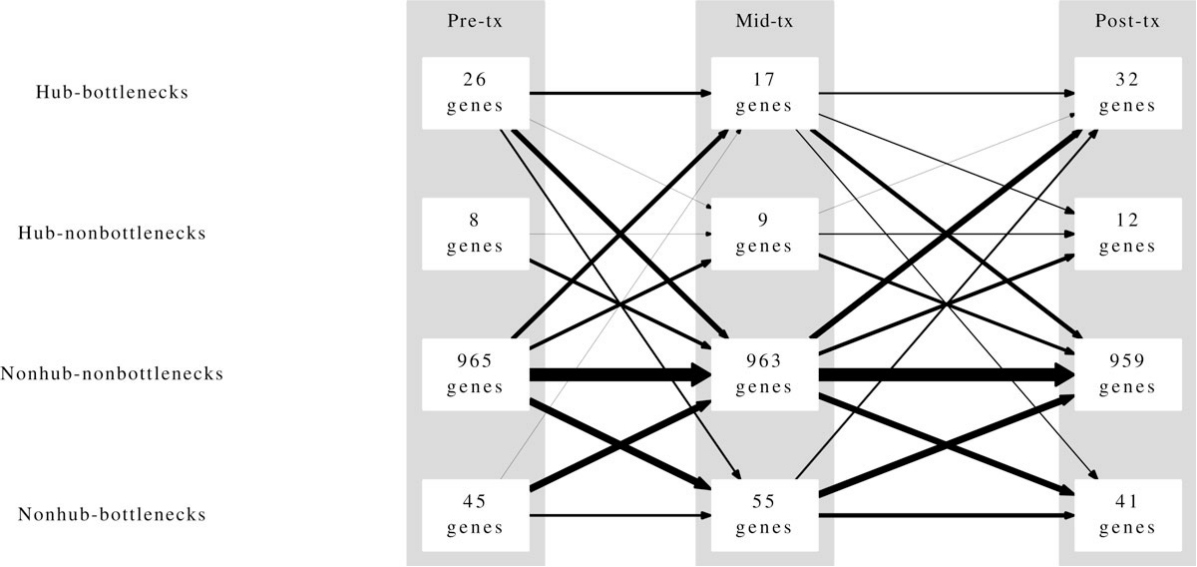
**Centrality classifications by treatment status**. Diagram illustrating the temporal positioning of genes within the coexpression network. Each row indicates a centrality category and each column indicates a time point. Arrows show how genes move between centrality categories over the course of letrozole treatment. Arrow thickness is proportional to the log of the number of genes being represented by a given arrow.

Of the 1044 differentially expressed genes, 852 or approximately 82%, are categorized as nonhub nonbottlenecks at all three time points (Figure 3). The remaining 18% of differentially expressed genes are holding prominent positions in at least one of the three networks. In most cases, a node categorized as a hub, a bottleneck, or both becomes a nonhub nonbottleneck gene at the next time point (Figure 4). In addition, most of the genes that become hubs, bottlenecks, or both were nonhub nonbottlenecks at the previous time point. This emphasizes that the relationships of individual genes are dynamically changing allowing nodes to cycle through positions of influence. As the network reacts to letrozole perturbation there is independent regulation of key genes in a time dependent manner.

Only one gene, ZFHX4, is classified into the same centrality category, a hub bottleneck, in all three networks. The ZFHX4 gene encodes a zinc-finger transcription factor that is upregulated by letrozole. According to the Human Protein Atlas there is consistent positive staining for ZFHX4 protein in the nucleus of breast tumor tissue samples and in at least two cell-lines derived from breast cancers, MCF-7, an ER^+ ^line, and SK-BR-3, an ER*^− ^*line [27,28]. The function of ZFHX4 is unknown but it has been predicted to participate in protein-protein interactions with androgen receptor (AR) and peroxisome proliferator-activated receptor gamma (PPARG) in beef cattle where it is associated with the regulation of puberty [29]. Additionally, ZFHX4 has been associated with neuronal and muscle differentiation in mice and interferon beta therapy response in MS patients [30,31]. As a stable hub bottleneck across our networks ZFHX4 likely plays a critical role in breast cancer as well.

There are five genes, BMP2, CYB5R3, DAB2, FAT4, and GTPBP4, that are consistently categorized as bottlenecks (either hub bottlenecks or nonhub bottlenecks). Briefly, BMP2 is a member of the TGF*β *superfamily and is upregulated by letrozole. It induces bone formation and is involved in osteoblast differentation and in conjunction with BMP7 it may prevent breast cancer metastases to the bone [32]. The signaling relationships between the ER and BMP2 pathways is well established [33]. Estrogen inihibits the expression of BMP2 through estrogen receptor signaling, a process that can be reversed with tamoxifen treatment. CYB5R3 has the largest gain in number of links following 90 days of letrozole treatment and it is upregulated by the drug. This gene has known functions in fatty acid metabolism, cholesterol biosynthesis and drug metabolism [34]. DAB2 functions as a mitogen responsive phosphoprotein, it is a putative tumor suppressor and it is upregulated by letrozole. It has been shown in breast cancer that the loss of DAB2 enables the epithelial-to-mesenchymal transition induced by TGF*β *signaling [35]. Notably, BMP2 and DAB2 are both used as stem cell markers. FAT4 encodes a tumor suppressor that is lost in a subset of breast cancer cell-lines and primary breast tumors [36]. FAT4 is upregulated by letrozole. GTPBP4 encodes a GTPase that is downregulated by letrozole. RNAi screening has revealed an interaction between GTPBP4 and p53 and the authors confirm that elevated levels of GTPBP4 in p53 WT breast tumors are inversely correlated with survival [37]. As enduring bottlenecks, these genes have links that maintain paths of information-flow throughout the networks.

One gene, CAV1, is consistently categorized as a hub (either a hub bottleneck or a hub nonbottleneck). CAV1 encodes a scaffolding protein that is a tumor suppressor candidate and it is upregulated by letrozole. CAV1 has been shown to target ER*α *to the cell membrane [38]. Extensive studies of CAV-1 in normal and tumor tissue have revealed its many roles in cell morphology, adhesion, remodeling of the tumor microenvironment, tumor cell invasion and metastic potential [39]. As an enduring hub, CAV1 plays a central role in maintaining coexpression network integrity.

Genes that have transiently high centrality scores are likely playing condition-dependent functional roles. There are 146 genes that are categorized as hubs, bottlenecks, or both in only one of the three networks. Briefly, the pre-treatment network has 49 of these genes including ID3, a gene that is upregulated by letrozole and involved in estrogen stimulated vascularization, and IGF1R a gene that is downregulated by letrozole and whose downregulation is associated with tamoxifen resistance in cell-lines and xenograft models [40,41]. The mid-treatment network has 50 of these genes including TACC1, a gene that is upregulated by letrozole and whose overexpression is associated with tamoxifen resistance, and RAD51 a gene that is downregulated by letrozole with a functional role in homologous recombination and known interactions with BRCA1 and BRCA2 [34,42,43]. The post-treatment network accounts for the remaining 47 genes in this group including PGR which is downregulated by letrozole and has a well-established role in hormone responsive cancers including breast cancer and TCF4 which induces osteoblast proliferation and differentiation and encodes a protein that physically interacts with ER*α *through the Wnt signaling pathway [44].

In addition, there are 39 genes that are hubs or bottlenecks at two of the three time points. A few of these are known estrogen responsive genes, for example, FOXO1 and KIAA0101. A complete list of differentially expressed genes with their centrality statuses are presented in Additional file 1.

**Figure 5:**
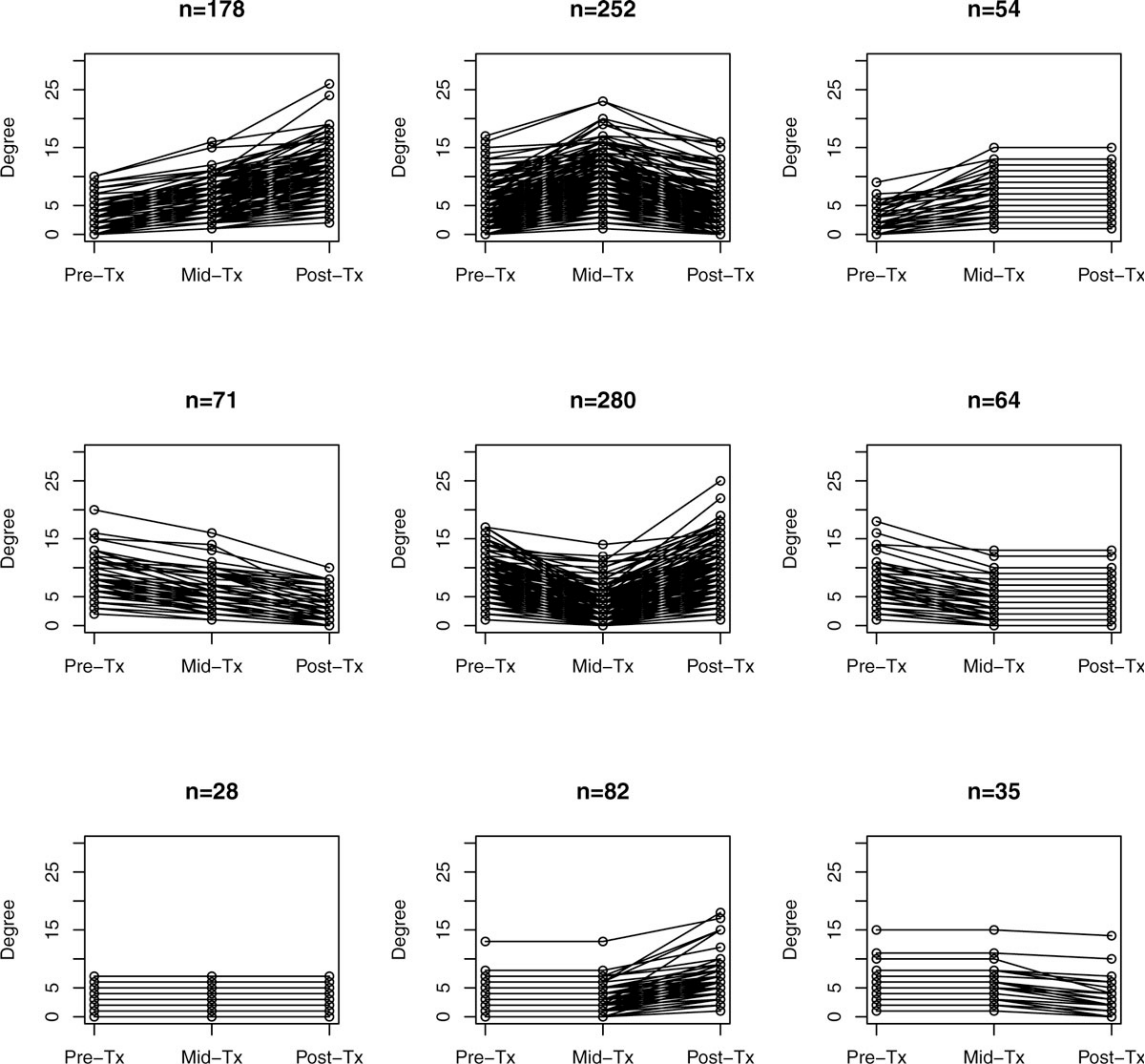
**Patterns of degree change. **Nodes may have an increase, decrease, or no change in degree as they adapt to the presence of letrozole. These plots illustrate the changes in degree of each node and the distribution of nodes across the nine possible patterns of change.

In order for nodes to have temporal influence they must either gain or lose enough links to change their own status, gain or lose one or more critical neighbors, or surrounding nodes must gain or lose enough links to change the status of a given node. Early response to letrozole treatment leads to the formation of additional relationships for the majority of genes (484/1044) (Figure 5). Although, the proportion of genes that have an initial loss of links is nearly as large (415/1044). The transition from early response to late response results in 540 and 358 genes gaining or losing links, respectively. Some nodes do have a consistent degree throughout the course of letrozole treatment however, stable degree is not a proxy for stable gene-gene relationships.

We counted the number of links that are conserved between both transitions from the pre-treatment to mid-treatment networks and from the mid-treatment to post-treatment networks. We find that approximately 20% of links are conserved between each of the time transitions meaning that 80% of gene-gene relationships are not stable throughout the course of letrozole treatment. This is consistent with a study of synthetic lethality in yeast which demonstrated that greater than 70% of the links representing epistatic relationships in a gene network following treatment with a DNA damaging agent are not present before-hand [45]. We show that coexpression networks are equally dynamic, continually rewiring in a condition dependent manner.

Partial correlation yields many false-negatives (i.e. not identifying relationships that are actually present in the data); however, the number of false positives (i.e. identifying relationships that are statistical artifacts) is neglible [17]. We are not concerned by the false negative links because our intention is not to reconstruct the entire gene regulatory network but instead to find similarities and differences between the condition specific networks. By using partial correlation to estimate coexpression we are confident that the links we identify are real and that the neighbors of each gene in each network are the optimal set of genes to explain the variation of a given gene within this data set.

The essentiality of hubs and bottlenecks has been debated in the literature with experimental evidence in support of both sides [25,26,46,47]. Here, we show that hubs and bottlenecks are transient in a time-dependent and condition-specific manner. Our results suggest that the underlying context determines the importance of each of these nodes. It is entirely feasible that nodes categorized as hubs and bottlenecks are only essential some of the time.

Due to the nature of coexpression all relationships are reciprocal so we cannot infer functional directions along the links. However, through literature validation we have shown that coexpression networks can provide a means of prioritizing genes that may play an especially important role as gene networks adapt to letrozole perturbation. As additional data measuring gene expression before and after letrozole treatment in vivo is collected and made available we will be able to validate this method and these results. Until then, our results can be used to generate testable hypotheses about letrozole's action or more generally about ER^+ ^breast cancer (e.g. the role of ZFHX4).

## Conclusions

Genomic data has the potential to reveal dynamic relationships between genes if analyzed in a way that respects the global set of interactions that underlie biological function. Network models can complement the more traditional differential expression analysis methods by revealing genes that have the potential to propagate information throughout entire gene networks changing gene-gene relationships along the way. In a letrozole perturbed breast tumor coexpression network we have identified key genes for modulating information flow at each of three treatment time points. Many of these genes are specific to either the pre- treatment, mid-treatment or post-treatment conditions which emphasizes the context-dependent assembly and dynamic nature of gene networks. By understanding how networks are rewired by letrozole treatment and which genes seem to mediate these effects we can begin to explore mechanisms of letrozole response or resistance.

## Competing interests

The authors declare that there are no competing interests.

## Authors' contributions

NP conceived of the study, performed the analyses and drafted the manuscript. JM participated in study design and helped to draft the manuscript. Both authors read and approved the final manuscript.

## Acknowledgements

This work was supported by NIH grants: LM010098, LM009012, AI59694.

## Declarations

The publication costs for this article were funded by JM.

This article has been published as part of *BMC Medical Genomics *Volume 6 Supplement 2, 2013: Selected articles from the Second Annual Translational Bioinformatics Conference (TBC 2012). The full contents of the supplement are available online at http://www.biomedcentral.com/bmcmedgenomics/supplements/6/S2.

## Supplementary Material

Additional file 1 **Differentially expressed genes and centrality status**. This file contains a table listing all of the 1044 differentially expressed genes. Cells containing a one indicate the gene has hub or bottleneck status at the indicated time point, otherwise the cell contains a zero. Click here for file
