# Supplementary material for: Key genes for modulating information flow play a temporal role as breast tumor coexpression networks are dynamically rewired by letrozole
Source: BMC Med Genomics. 2013 May 7;6(Suppl 2):S2. doi: 10.1186/1755-8794-6-S2-S2 (PMC3654875; doi:10.1186/1755-8794-6-S2-S2)
Supplement: Additional file 1 — Differentially expressed genes and centrality status. This file contains a table listing all of the 1044 differentially expressed genes. Cells containing a one indicate the gene has hub or bottleneck status at the indicated time point, otherwise the cell contains a zero. [file 1755-8794-6-S2-S2-S1.pdf]

Genes

Entrez Gene ID	Gene Symbol	Hub			Bottleneck		
		Pre-tx	Mid-tx	Post-tx	Pre-tx	Mid-tx	Post-tx
2	A2M	0	1	0	0	1	0
79719	AAGAB	0	0	0	0	0	0
18	ABAT	0	0	0	0	0	0
21	ABCA3	0	0	0	0	0	0
23460	ABCA6	0	0	0	0	0	0
10351	ABCA8	0	0	0	0	0	0
5243	ABCB1	0	0	0	0	0	0
10257	ABCC4	0	0	0	0	0	0
5825	ABCD3	0	0	0	0	0	0
25	ABL1	0	0	0	0	0	0
39	ACAT2	0	0	0	0	0	0
8309	ACOX2	0	0	0	0	0	0
52	ACP1	0	0	0	0	0	0
59	ACTA2	0	0	0	0	0	0
72	ACTG2	0	0	0	0	0	0
86	ACTL6A	0	0	0	0	0	0
8038	ADAM12	0	0	0	0	0	0
9510	ADAMTS1	0	0	0	0	0	0
11096	ADAMTS5	0	0	1	0	0	1
104	ADARB1	0	0	0	0	0	0
107	ADCY1	0	0	0	0	0	0
120	ADD3	0	0	0	0	0	0
150	ADRA2A	0	0	0	0	0	0
154	ADRB2	0	0	0	0	0	0
165	AEBP1	1	0	0	0	0	0
8540	AGPS	0	0	0	0	0	0
10551	AGR2	0	0	0	0	0	0
113146	AHNAK2	0	0	0	0	0	0
7965	AIMP2	0	0	0	0	0	0
9590	AKAP12	0	0	1	0	0	0
231	AKR1B1	0	0	0	1	0	0
10000	AKT3	0	0	0	0	0	0
216	ALDH1A1	0	0	0	0	0	0
51421	AMOTL2	0	0	0	0	0	0
23452	ANGPTL2	0	0	1	0	0	0
287	ANK2	0	0	0	1	1	0
22881	ANKRD6	0	0	0	0	0	0
301	ANXA1	0	1	0	0	0	0
311	ANXA11	0	0	0	0	0	0
308	ANXA5	0	0	0	0	0	0
8416	ANXA9	0	0	0	0	0	0
8639	AOC3	0	0	0	0	0	0
1174	AP1S1	0	0	0	0	0	0
378708	APITD1	0	0	0	0	0	0
187	APLNR	0	0	0	0	0	0
9582	APOBEC3B	0	0	0	0	0	0
54840	APTX	0	0	0	0	0	0
358	AQP1	0	0	0	0	0	0
392	ARHGAP1	0	0	0	0	0	0

55114	ARHGAP17	0	0	0	0	0	0
9639	ARHGEF10	0	0	0	0	0	0
55701	ARHGEF40	0	0	0	0	0	0
84159	ARID5B	0	0	0	0	0	0
403	ARL3	0	0	0	0	0	0
10123	ARL4C	0	0	0	0	0	0
23204	ARL6IP1	0	0	0	0	0	0
81873	ARPC5L	0	0	0	0	0	1
55616	ASAP3	0	0	0	0	0	0
79754	ASB13	0	0	0	0	0	0
429	ASCL1	0	0	0	0	1	0
259266	ASPM	0	0	0	0	0	0
29028	ATAD2	0	0	0	0	0	1
467	ATF3	0	0	0	0	0	0
471	ATIC	1	0	0	1	0	0
482	ATP1B2	0	0	0	0	0	0
509	ATP5C1	0	0	0	0	1	0
27109	ATP5S	0	0	0	0	0	0
533	ATP6V0B	0	0	0	0	0	0
51382	ATP6V1D	0	0	0	0	0	0
6310	ATXN1	0	0	0	0	0	0
4287	ATXN3	0	0	0	0	0	0
6790	AURKA	0	0	0	0	0	0
9212	AURKB	0	0	0	0	0	0
558	AXL	0	0	0	0	0	0
60468	BACH2	0	0	0	0	1	0
56647	BCCIP	0	0	0	0	0	0
633	BGN	0	0	0	0	1	0
79365	BHLHE41	0	0	0	0	0	0
274	BIN1	0	0	0	0	1	0
332	BIRC5	0	0	0	0	1	1
644	BLVRA	0	0	0	0	0	0
650	BMP2	0	0	1	1	1	1
652	BMP4	0	0	0	0	0	0
54796	BNC2	0	0	0	0	0	0
55299	BRIX1	0	0	0	0	0	0
7832	BTG2	0	0	0	0	0	0
11120	BTN2A1	0	0	0	0	0	0
10384	BTN3A3	0	0	0	0	0	0
701	BUB1B	0	0	0	0	0	0
9184	BUB3	0	0	0	0	0	1
28969	BZW2	0	0	0	0	0	0
11067	C10orf10	0	0	0	0	0	0
83638	C11orf68	0	0	0	0	0	0
56935	C11orf75	0	0	0	0	0	0
79697	C14orf169	0	0	0	0	0	0
9556	C14orf2	0	0	0	0	0	0
114897	C1QTNF1	0	0	0	0	0	0
715	C1R	0	1	1	0	1	0
716	C1S	1	0	0	0	0	0
54994	C20orf11	0	0	0	0	0	0
25966	C2CD2	0	0	0	0	0	0

388969	C2orf68	0	0	0	0	0	0
718	C3	0	0	0	0	0	0
79624	C6orf211	0	0	0	0	0	0
730	C7	0	0	0	0	0	0
55744	C7orf44	0	0	0	0	0	0
771	CA12	0	0	0	0	0	0
27101	CACYBP	0	0	0	0	0	1
800	CALD1	0	0	0	0	0	0
51063	CALHM2	0	0	0	0	0	0
23125	CAMTA2	0	0	0	0	0	0
10486	CAP2	0	0	0	0	0	0
65981	CAPRIN2	0	0	0	0	0	0
857	CAV1	1	1	1	0	0	1
868	CBLB	0	0	0	0	0	0
23624	CBLC	0	0	0	0	0	0
11335	CBX3	0	0	0	1	0	0
63933	CCDC90A	0	0	0	0	0	0
80212	CCDC92	0	0	0	0	0	0
6351	CCL4	0	0	0	0	0	0
890	CCNA2	0	0	0	0	0	0
891	CCNB1	0	0	0	1	0	0
9133	CCNB2	0	0	0	0	0	0
595	CCND1	0	0	0	0	0	0
894	CCND2	0	0	0	0	0	0
10576	CCT2	0	0	0	0	0	0
7203	CCT3	0	0	0	1	0	0
22948	CCT5	0	0	0	0	0	0
908	CCT6A	0	0	0	0	0	1
4345	CD200	1	0	0	1	0	0
57124	CD248	0	0	0	0	0	0
947	CD34	0	0	0	0	0	0
972	CD74	0	0	0	0	0	0
9308	CD83	0	0	0	0	0	0
22918	CD93	0	0	1	0	0	1
4267	CD99	0	0	0	0	0	0
8555	CDC14B	0	0	0	0	0	0
991	CDC20	0	0	0	0	0	0
993	CDC25A	0	0	0	0	0	0
990	CDC6	0	0	0	0	0	0
55038	CDCA4	0	0	0	0	0	0
55143	CDCA8	0	0	0	0	1	0
1009	CDH11	0	0	0	0	0	0
1003	CDH5	0	0	0	0	0	0
983	CDK1	0	0	0	0	0	0
1028	CDKN1C	0	0	0	0	0	0
1033	CDKN3	0	0	0	0	0	0
51816	CECR1	0	0	0	0	0	0
27440	CECR5	0	0	0	0	0	0
10659	CELF2	1	1	0	1	1	0
9620	CELSR1	0	0	0	0	0	0
1952	CELSR2	0	0	0	0	0	0
1058	CENPA	0	0	0	0	1	0

1063	CENPF	0	0	0	0	0	0
79019	CENPM	0	0	0	0	0	0
55839	CENPN	0	0	0	1	0	0
55165	CEP55	0	0	0	0	0	0
23177	CEP68	0	0	0	0	0	0
1069	CETN2	0	0	0	0	0	0
3075	CFH	0	0	0	0	0	0
3080	CFHR2	0	0	0	0	0	0
3426	CFI	0	0	0	0	0	0
9023	CH25H	0	0	0	0	0	0
1111	CHEK1	0	0	0	0	0	0
1118	CHIT1	0	0	0	0	0	0
26973	CHORDC1	0	0	0	0	0	0
91851	CHRDL1	0	0	0	0	0	0
64377	CHST8	0	0	0	0	0	0
8483	CILP	0	0	0	0	0	0
51550	CINP	0	0	0	0	0	0
26586	CKAP2	0	0	0	0	1	0
1164	CKS2	0	0	0	0	0	0
7122	CLDN5	0	0	1	0	0	1
1366	CLDN7	0	0	0	0	0	0
6320	CLEC11A	0	0	0	0	0	0
7123	CLEC3B	0	0	0	0	0	0
25999	CLIP3	0	0	0	1	1	0
1191	CLU	0	0	0	0	0	0
8418	CMAHP	0	0	0	0	0	0
7373	COL14A1	0	0	0	0	0	0
1306	COL15A1	0	0	0	0	0	0
1307	COL16A1	1	0	1	1	0	1
80781	COL18A1	0	0	0	0	0	0
1277	COL1A1	0	0	0	0	0	0
1278	COL1A2	0	0	0	0	0	0
81578	COL21A1	0	0	0	0	0	0
1281	COL3A1	0	0	0	0	0	0
1289	COL5A1	0	1	0	0	1	0
50509	COL5A3	0	0	0	0	0	0
1291	COL6A1	0	0	0	0	0	0
1292	COL6A2	0	1	1	0	1	0
1293	COL6A3	0	0	0	0	0	0
1296	COL8A2	0	0	0	0	0	0
81035	COLEC12	0	0	0	0	0	0
51226	COPZ2	0	0	0	0	0	0
27235	COQ2	0	0	0	0	0	0
1345	COX6C	0	0	0	0	0	0
1347	COX7A2	0	0	0	0	0	0
1368	CPM	0	0	0	0	0	0
1376	CPT2	0	0	0	0	0	0
54504	CPVL	0	0	0	0	0	0
8532	CPZ	0	0	0	0	0	0
83716	CRISPLD2	0	1	0	0	1	0
84809	CROCCP2	0	0	0	0	0	0
64784	CRTC3	0	0	0	0	0	0

1408	CRY2	0	0	0	0	0	0
1410	CRYAB	0	0	0	0	0	0
1434	CSE1L	1	0	0	1	0	0
1436	CSF1R	0	0	0	0	1	1
1466	CSRP2	0	0	0	0	0	0
1474	CST6	0	0	0	0	0	0
8530	CST7	0	0	0	0	0	0
58190	CTDSP1	0	0	0	0	0	0
1490	CTGF	0	0	0	0	0	0
1503	CTPS1	0	0	0	0	0	0
1513	CTSK	0	0	0	0	0	0
1519	CTSO	0	0	0	0	0	0
6376	CX3CL1	0	0	0	0	0	0
6387	CXCL12	0	0	1	0	0	1
63932	CXorf56	0	0	0	0	0	0
1727	CYB5R3	0	1	1	1	1	1
1556	CYP2B7P1	0	0	0	0	0	0
3491	CYR61	0	0	0	0	0	0
23500	DAAM2	0	0	0	0	0	0
1601	DAB2	1	1	0	1	1	1
2532	DARC	0	0	0	0	1	0
8642	DCHS1	0	0	0	0	0	0
1634	DCN	1	0	0	0	0	0
55802	DCP1A	0	0	0	0	0	0
79077	DCTPP1	0	0	0	0	0	0
9188	DDX21	0	0	0	0	0	0
1654	DDX3X	0	0	0	0	0	0
23586	DDX58	0	0	0	0	0	0
8562	DENR	0	0	0	0	0	0
27351	DESI1	0	0	0	0	0	0
1687	DFNA5	0	0	0	0	0	0
1717	DHCR7	0	0	0	0	0	0
1725	DHPS	0	0	0	0	0	0
9249	DHRS3	0	0	0	0	0	0
10170	DHRS9	0	0	0	0	0	0
56616	DIABLO	0	0	0	0	0	0
22982	DIP2C	0	0	0	0	0	0
1736	DKC1	0	0	0	0	1	0
27123	DKK2	0	0	0	0	0	0
27122	DKK3	0	0	0	0	0	0
10395	DLC1	0	0	0	0	0	0
9787	DLGAP5	0	0	0	0	0	0
56521	DNAJC12	0	0	0	0	0	0
1776	DNASE1L3	0	0	0	0	0	0
1759	DNM1	0	0	0	0	0	0
10059	DNM1L	0	0	0	0	0	0
1805	DPT	0	0	0	0	0	0
1808	DPYSL2	0	0	0	0	0	1
1809	DPYSL3	0	1	0	0	1	0
4733	DRG1	0	0	0	0	0	0
79075	DSCC1	0	0	0	0	0	0
79980	DSN1	0	0	0	0	0	0

667	DST	0	0	0	0	0	0
51514	DTL	0	0	0	0	0	0
1841	DTYMK	0	0	0	0	0	0
1843	DUSP1	0	0	0	0	0	0
11072	DUSP14	0	0	0	0	0	0
6993	DYNLT1	0	0	0	0	0	0
6990	DYNLT3	0	0	0	0	0	0
22873	DZIP1	0	0	0	0	0	0
10969	EBNA1BP2	0	0	0	0	0	0
1632	ECI1	1	0	0	1	0	0
1842	ECM2	0	0	0	0	0	0
1894	ECT2	0	0	0	0	1	0
9521	EEF1E1	0	0	0	0	0	0
90141	EFCAB11	0	0	0	0	0	0
30008	EFEMP2	1	1	0	1	0	0
25975	EGFL6	0	0	0	0	0	0
1958	EGR1	0	0	0	0	0	0
1959	EGR2	0	0	0	0	0	0
30846	EHD2	0	0	1	1	0	1
8894	EIF2S2	0	0	0	0	0	0
2000	ELF4	0	0	0	0	0	0
80237	ELL3	0	0	0	0	0	0
2006	ELN	0	0	0	0	0	0
54898	ELOVL2	0	0	0	0	0	0
60481	ELOVL5	0	0	0	0	0	0
64123	ELTD1	0	0	0	0	0	0
11117	EMILIN1	0	0	0	0	0	0
2009	EML1	0	0	0	0	0	0
2012	EMP1	0	0	0	0	0	0
2018	EMX2	0	0	0	0	0	0
58478	ENOPH1	0	0	0	0	0	1
5168	ENPP2	0	0	0	0	0	0
2034	EPAS1	0	0	0	1	0	0
2037	EPB41L2	0	0	0	0	0	0
4072	EPCAM	0	0	1	0	0	1
1969	EPHA2	0	0	0	0	0	0
2059	EPS8	0	0	0	0	0	0
2078	ERG	0	0	0	0	0	0
2079	ERH	0	0	0	0	0	0
9700	ESPL1	0	0	0	0	0	0
2099	ESR1	0	0	0	0	0	0
55224	ETNK2	0	0	0	0	0	0
2114	ETS2	0	0	0	0	0	0
56915	EXOSC5	0	0	0	0	0	0
2146	EZH2	0	0	0	0	0	0
2161	F12	0	0	0	0	0	0
11170	FAM107A	0	0	0	0	0	1
92689	FAM114A1	0	0	0	0	0	0
81558	FAM117A	0	0	0	0	0	0
8933	FAM127A	0	0	0	0	0	0
10144	FAM13A	0	0	0	0	0	0
25854	FAM149A	0	0	0	0	0	0

51016	FAM158A	0	0	0	0	0	0
221061	FAM171A1	0	0	0	0	0	0
23272	FAM208A	0	0	0	0	0	0
54906	FAM208B	0	0	0	0	0	0
55603	FAM46A	0	0	0	0	0	0
55793	FAM63A	0	0	0	0	0	0
54478	FAM64A	0	0	0	0	0	0
79567	FAM65A	0	0	0	0	1	0
51115	FAM82B	0	0	0	0	0	0
2189	FANCG	0	0	0	0	0	0
55215	FANCI	0	0	0	0	0	0
2191	FAP	0	0	0	0	0	0
2195	FAT1	0	0	0	0	0	0
79633	FAT4	1	1	0	1	1	1
2192	FBLN1	0	0	1	0	0	0
2199	FBLN2	0	1	0	0	0	0
10516	FBLN5	0	0	0	0	0	0
115290	FBXO17	0	0	0	0	0	0
2205	FCER1A	0	0	0	0	0	0
2237	FEN1	0	0	0	0	0	0
10979	FERMT2	0	0	1	0	0	0
9638	FEZ1	0	0	0	0	0	0
2254	FGF9	0	0	0	0	0	0
11116	FGFR1OP	0	0	0	0	0	0
2261	FGFR3	0	0	0	0	0	0
2273	FHL1	0	0	0	0	0	0
80206	FHOD3	0	0	0	0	0	0
55033	FKBP14	0	0	0	0	0	0
2287	FKBP3	0	0	0	0	0	0
2288	FKBP4	0	0	0	0	0	0
2318	FLNC	0	0	0	0	0	0
23768	FLRT2	0	0	0	0	0	0
2331	FMOD	0	0	0	0	0	0
23048	FNBP1	0	0	0	0	0	0
2350	FOLR2	0	0	0	0	1	1
2353	FOS	0	0	0	0	0	0
2354	FOSB	0	0	0	0	0	0
2296	FOXC1	0	0	0	0	0	0
2305	FOXM1	0	0	0	0	0	0
1112	FOXN3	0	0	0	0	0	0
2308	FOXO1	0	0	1	1	0	1
2487	FRZB	0	0	0	0	0	0
11167	FSTL1	0	1	1	0	1	1
2517	FUCA1	0	0	0	0	0	0
26515	FXC1	0	0	0	0	0	0
5348	FXYD1	0	0	0	0	0	0
53827	FXYD5	0	0	0	0	0	0
53826	FXYD6	0	0	0	0	0	0
2534	FYN	0	0	0	0	1	0
8322	FZD4	0	0	0	0	0	0
79695	GALNT12	0	0	0	0	0	0
2619	GAS1	0	0	0	0	0	0

2621	GAS6	0	0	0	0	0	0
8522	GAS7	1	0	0	1	0	0
81544	GDPD5	0	0	0	0	0	0
2669	GEM	0	0	0	0	0	0
79833	GEMIN6	0	0	0	0	1	0
9945	GFPT2	0	0	0	0	1	0
2674	GFRA1	0	0	0	0	0	0
8836	GGH	0	0	0	0	0	0
9837	GINS1	0	0	0	0	0	0
51659	GINS2	0	0	0	0	0	0
64785	GINS3	0	0	0	0	0	0
54810	GIPC2	0	0	0	0	0	1
2701	GJA4	0	0	0	0	0	0
2717	GLA	0	0	0	0	1	0
2734	GLG1	0	0	0	0	0	0
51022	GLRX2	0	0	0	0	0	0
10539	GLRX3	0	0	0	0	0	0
83468	GLT8D2	0	0	0	0	0	0
2752	GLUL	0	0	0	0	0	0
2764	GMFB	0	0	0	0	0	0
51053	GMNN	0	0	0	0	0	0
2791	GNG11	0	0	0	0	1	0
27333	GOLIM4	0	0	0	0	0	0
64689	GORASP1	0	0	0	0	0	0
2806	GOT2	0	0	0	0	0	0
10457	GPNMB	0	0	0	0	0	0
221395	GPR116	0	0	0	0	0	0
25960	GPR124	0	0	1	0	0	1
27202	GPR77	0	0	0	0	0	0
9737	GPRASP1	0	0	0	0	0	0
9687	GREB1	0	0	0	0	0	0
2869	GRK5	0	0	0	0	0	0
80273	GRPEL1	0	0	0	0	0	0
2935	GSPT1	0	0	0	0	0	0
2949	GSTM5	0	0	0	0	0	0
404672	GTF2H5	0	0	1	0	0	1
23560	GTPBP4	1	0	0	1	1	1
2982	GUCY1A3	0	0	0	0	0	0
60558	GUF1	0	0	0	0	0	0
51454	GULP1	0	0	0	0	0	0
3014	H2AFX	0	0	0	0	0	0
3015	H2AFZ	0	0	0	1	0	0
10456	HAX1	0	0	0	0	0	0
3043	HBB	0	0	0	0	0	0
51696	HECA	0	0	0	0	0	0
57493	HEG1	0	0	0	0	0	1
8335	HIST1H2AB	0	0	0	0	0	0
85236	HIST1H2BK	0	0	0	0	0	1
8351	HIST1H3D	0	0	0	0	0	0
8366	HIST1H4B	0	0	0	0	0	0
3097	HIVEP2	0	0	0	0	0	0
55355	HJURP	0	0	0	1	0	0

3108	HLA-DMA	0	0	0	0	0	0
3131	HLF	0	0	0	1	0	0
6596	HLTF	0	0	0	0	0	0
3145	HMBS	0	0	0	1	0	0
3148	HMGB2	0	0	0	0	0	0
3149	HMGB3	0	0	0	0	0	0
3161	HMMR	1	0	0	1	0	1
51155	HN1	0	0	0	1	0	0
90861	HN1L	0	0	0	0	0	0
3182	HNRNPAB	0	0	0	0	0	0
51361	HOOK1	0	0	0	0	0	0
3206	HOXA10	0	0	0	0	0	0
3202	HOXA5	0	0	0	0	0	0
3223	HOXC6	0	0	0	0	0	0
3251	HPRT1	0	0	0	0	0	0
9956	HS3ST2	0	0	0	0	0	0
51170	HSD17B11	0	0	0	0	0	0
6782	HSPA13	0	0	0	0	0	0
51668	HSPB11	0	0	0	1	0	0
3316	HSPB2	0	0	0	1	0	0
26353	HSPB8	0	0	0	0	0	0
3357	HTR2B	0	0	0	0	0	0
5654	HTRA1	0	0	0	0	0	0
3376	IARS	0	0	0	0	0	0
3384	ICAM2	0	0	0	0	0	0
3397	ID1	0	0	0	0	0	0
3399	ID3	0	0	0	1	0	0
3400	ID4	0	0	0	0	0	0
9592	IER2	0	0	0	0	0	0
25900	IFFO1	0	0	0	0	0	0
2537	IFI6	0	0	0	0	0	0
3434	IFIT1	0	0	0	0	0	0
3479	IGF1	0	1	0	0	0	0
3480	IGF1R	0	0	0	1	0	0
3489	IGFBP6	0	0	1	0	0	1
3590	IL11RA	0	0	0	0	0	0
55540	IL17RB	0	0	0	0	0	0
3554	IL1R1	0	0	0	0	0	0
90865	IL33	0	0	0	0	0	0
3572	IL6ST	0	0	0	0	0	0
3625	INHBB	0	0	0	0	0	0
8821	INPP4B	0	0	0	0	0	0
9922	IQSEC1	0	0	0	0	0	0
3667	IRS1	0	0	0	0	0	0
9636	ISG15	0	0	0	0	0	0
3671	ISLR	0	0	0	0	0	0
3679	ITGA7	0	0	0	0	0	0
3682	ITGAE	0	0	0	0	0	0
23421	ITGB3BP	0	0	0	0	0	0
80760	ITIH5	0	0	0	0	1	0
9452	ITM2A	1	0	0	0	0	0
3704	ITPA	0	0	0	0	0	0

3707	ITPKB	0	0	0	0	0	0
6453	ITSN1	0	1	1	0	0	0
58494	JAM2	1	0	0	1	0	0
83700	JAM3	0	0	0	1	0	0
3725	JUN	0	0	0	0	0	0
3726	JUNB	0	0	0	0	0	0
25959	KANK2	0	1	1	0	0	0
8850	KAT2B	0	0	0	0	0	0
60598	KCNK15	0	0	0	0	0	0
3778	KCNMA1	0	0	0	0	0	0
27345	KCNMB4	0	0	0	0	0	0
115207	KCTD12	1	0	1	1	0	1
23030	KDM4B	0	0	0	0	0	0
11081	KERA	0	0	0	0	0	0
10656	KHDRBS3	0	0	0	0	0	0
9768	KIAA0101	0	0	0	0	1	1
23199	KIAA0182	0	0	0	0	0	0
9710	KIAA0355	0	0	0	0	0	0
9692	KIAA0391	0	0	0	0	0	0
57608	KIAA1462	0	0	0	0	0	0
3832	KIF11	0	0	0	0	0	0
9928	KIF14	0	0	0	0	0	0
56992	KIF15	0	0	0	0	0	0
10112	KIF20A	0	0	0	0	0	0
9493	KIF23	0	0	0	0	0	0
11004	KIF2C	0	0	0	0	0	0
24137	KIF4A	0	0	0	0	0	0
3833	KIFC1	0	0	0	0	0	1
3815	KIT	0	0	0	0	0	0
10365	KLF2	0	0	0	0	0	0
1316	KLF6	0	0	0	0	0	0
687	KLF9	0	0	0	0	0	0
26249	KLHL3	0	0	0	0	1	0
3838	KPNA2	0	0	0	0	0	0
8270	LAGE3	0	0	0	0	0	0
3908	LAMA2	0	0	0	0	1	1
3912	LAMB1	0	0	0	0	0	0
55353	LAPTM4B	0	0	0	0	0	0
55323	LARP6	0	0	0	0	0	0
9079	LDB2	0	0	0	0	0	0
5641	LGMN	0	0	0	0	0	0
10186	LHFP	0	0	0	0	0	0
26468	LHX6	0	0	0	0	0	0
51474	LIMA1	0	0	0	0	0	0
22998	LIMCH1	0	0	0	0	0	0
55679	LIMS2	0	0	0	0	0	0
79686	LINC00341	0	0	1	0	0	1
150759	LINC00342	0	0	0	0	0	0
4001	LMNB1	0	0	0	0	0	0
4005	LMO2	0	0	0	0	0	0
25802	LMOD1	0	0	0	0	0	0
100505487	LOC100505487	0	0	0	0	0	0

100507314	LOC100507314	0	0	0	0	0	0
728392	LOC728392	0	0	0	0	0	0
4015	LOX	0	0	0	0	0	0
1902	LPAR1	0	0	0	0	0	0
23266	LPHN2	0	0	0	0	0	0
23175	LPIN1	0	0	0	0	0	0
4035	LRP1	0	0	0	0	0	0
10234	LRRC17	0	0	0	0	0	0
2615	LRRC32	0	0	0	0	0	0
27258	LSM3	0	0	0	0	0	0
25804	LSM4	0	0	0	0	0	0
4046	LSP1	0	1	0	0	1	0
7798	LUZP1	0	0	0	0	0	0
4085	MAD2L1	0	0	0	1	0	0
9587	MAD2L1BP	0	0	0	0	0	0
4094	MAF	0	0	0	0	0	0
9935	MAFB	0	0	0	0	0	0
10916	MAGED2	0	0	0	0	0	0
54551	MAGEL2	0	0	0	0	0	0
55110	MAGOHB	0	0	0	0	0	0
7873	MANF	0	0	0	0	0	0
4131	MAP1B	0	0	0	0	0	0
4215	MAP3K3	0	0	0	0	0	0
9448	MAP4K4	0	0	0	0	0	1
9053	MAP7	0	0	0	0	0	0
9261	MAPKAPK2	0	0	0	0	0	0
10982	MAPRE2	0	0	0	0	0	0
4137	MAPT	0	0	0	0	0	0
54708	MARCH5	0	0	0	0	0	0
4162	MCAM	0	0	1	0	0	1
4171	MCM2	0	0	0	0	0	0
4172	MCM3	0	0	0	0	0	0
4173	MCM4	0	0	0	0	0	0
4175	MCM6	0	0	0	0	0	0
2122	MECOM	0	0	0	0	0	0
23389	MED13L	0	0	0	0	0	0
4205	MEF2A	0	0	0	0	0	0
4208	MEF2C	0	0	0	0	0	0
55384	MEG3	0	0	0	0	1	0
4211	MEIS1	0	0	0	0	0	0
4212	MEIS2	0	1	0	0	0	0
9833	MELK	0	0	0	0	0	0
4222	MEOX1	0	0	0	0	0	0
4223	MEOX2	0	0	0	0	0	0
25840	METTL7A	0	0	0	0	0	0
4239	MFAP4	0	0	0	0	0	0
4242	MFNG	0	0	0	0	0	0
4281	MID1	1	0	0	1	0	1
4286	MITF	0	0	0	0	0	0
79682	MLF1IP	0	0	0	0	0	0
22877	MLXIP	0	0	0	0	0	0
4311	MME	1	0	0	1	1	0

4313	MMP2	0	0	0	0	0	0
22915	MMRN1	0	0	0	0	0	0
79812	MMRN2	0	0	0	0	0	0
4330	MN1	0	0	0	0	0	0
55233	MOB1A	0	0	0	0	0	0
55034	MOCOS	0	0	0	0	0	0
4337	MOCS1	0	0	0	0	0	0
26002	MOXD1	0	0	0	0	0	0
4354	MPP1	0	0	0	0	0	0
9902	MRC2	1	0	0	1	0	0
55686	MREG	0	0	0	0	0	0
78988	MRP63	0	0	0	0	0	0
65003	MRPL11	0	0	0	0	0	0
6182	MRPL12	0	0	0	0	0	0
28998	MRPL13	0	0	0	1	0	0
63875	MRPL17	1	0	0	1	1	0
11222	MRPL3	0	0	0	0	0	0
28977	MRPL42	0	0	0	0	0	0
6183	MRPS12	0	0	0	0	0	0
51373	MRPS17	0	0	0	1	0	0
10884	MRPS30	0	0	0	0	0	0
60488	MRPS35	0	0	0	0	0	0
51081	MRPS7	0	0	0	0	0	0
2206	MS4A2	0	0	0	0	0	0
2956	MSH6	0	0	0	0	0	0
6307	MSMO1	0	0	0	0	0	0
4478	MSN	0	0	0	0	0	0
4487	MSX1	0	0	0	0	0	0
23788	MTCH2	0	0	0	0	0	0
10797	MTHFD2	0	0	0	0	0	0
4528	MTIF2	0	0	0	0	0	0
9633	MTL5	0	0	0	0	0	0
10651	MTX2	0	0	0	0	0	0
25878	MXRA5	0	0	0	0	0	0
54587	MXRA8	1	0	0	0	0	0
4602	MYB	0	0	0	0	0	0
4603	MYBL1	0	0	0	0	0	0
4628	MYH10	0	0	0	0	0	0
4629	MYH11	0	0	0	0	0	0
10398	MYL9	0	0	0	0	0	0
4638	MYLK	0	0	1	0	0	1
4642	MYO1D	0	0	0	0	0	0
8736	MYOM1	0	0	0	0	0	0
25924	MYRIP	0	0	0	0	0	0
653784	MZT2A	0	0	0	0	0	0
80097	MZT2B	0	0	0	0	0	0
29104	N6AMT1	0	0	0	0	0	0
4675	NAP1L3	0	0	0	0	0	0
9	NAT1	0	0	0	0	0	0
64151	NCAPG	0	0	0	0	0	0
54892	NCAPG2	0	0	0	0	0	0
23397	NCAPH	0	0	1	0	0	1

8648	NCOA1	0	0	0	0	0	0
10403	NDC80	0	0	0	0	0	0
4692	NDN	0	0	1	1	0	1
4701	NDUFA7	0	1	0	0	1	0
4706	NDUFAB1	0	0	0	0	0	0
29078	NDUFAF4	0	0	0	0	0	0
54539	NDUFB11	0	0	0	0	0	0
4709	NDUFB3	0	0	0	0	0	0
4747	NEFL	0	0	0	1	0	0
4751	NEK2	0	0	0	0	0	0
10763	NES	0	0	0	0	0	0
4781	NFIB	0	0	0	0	0	0
55651	NHP2	1	0	1	1	0	1
4811	NID1	0	0	0	0	0	0
51388	NIP7	0	0	0	0	0	0
8508	NIPSNAP1	0	0	0	0	0	0
11188	NISCH	0	0	0	0	0	0
56954	NIT2	0	0	0	0	0	0
79570	NKAIN1	0	0	0	0	0	0
4830	NME1	0	0	1	1	0	1
8382	NME5	0	0	0	0	0	0
10874	NMU	0	0	0	0	0	0
26155	NOC2L	0	0	0	0	0	0
25926	NOL11	0	0	0	0	0	0
51491	NOP16	0	0	0	0	0	0
4856	NOV	0	0	0	0	0	0
4869	NPM1	0	0	0	0	0	0
4882	NPR2	0	0	0	0	0	0
4885	NPTX2	0	0	0	0	0	0
4886	NPY1R	0	0	0	0	0	0
7026	NR2F2	0	0	0	0	0	0
2908	NR3C1	0	0	0	0	0	0
4306	NR3C2	0	0	0	0	0	0
3164	NR4A1	0	0	0	0	0	0
51299	NRN1	0	0	0	1	0	0
8829	NRP1	0	0	0	0	0	0
4907	NT5E	0	0	0	0	0	0
11051	NUDT21	0	0	0	0	0	0
9631	NUP155	0	0	0	0	0	0
79023	NUP37	0	0	0	0	1	1
348995	NUP43	0	0	0	0	0	0
4928	NUP98	0	0	0	0	0	0
51203	NUSAP1	0	0	0	0	0	0
10482	NXF1	0	0	0	1	0	0
64359	NXN	0	0	0	0	0	0
4969	OGN	0	0	0	0	0	0
11339	OIP5	0	0	0	0	0	0
29789	OLA1	0	0	0	0	0	1
283298	OLFML1	1	1	0	1	1	0
25903	OLFML2B	0	0	0	0	0	0
56944	OLFML3	0	0	0	0	0	0
10133	OPTN	0	0	0	0	0	0

4999	ORC2	0	0	0	0	0	0
23594	ORC6	0	0	0	0	0	0
29095	ORMDL2	0	0	0	0	0	0
116039	OSR2	0	0	0	0	0	0
58495	OVOL2	0	0	0	0	0	0
54995	OXSM	0	0	0	0	0	0
53829	P2RY13	0	0	0	0	0	0
23241	PACS2	0	0	0	0	0	0
5050	PAFAH1B3	0	0	1	0	1	1
10606	PAICS	0	0	0	0	0	0
54873	PALMD	0	0	0	0	0	0
142	PARP1	0	0	0	0	0	0
55010	PARPBP	0	0	0	0	0	0
55742	PARVA	0	0	0	0	0	0
55872	PBK	0	0	0	0	0	0
5110	PCMT1	0	0	0	0	1	0
5111	PCNA	0	0	0	0	0	0
5118	PCOLCE	1	0	1	1	0	0
5125	PCSK5	0	0	0	0	0	0
5138	PDE2A	0	0	0	0	0	1
80310	PDGFD	0	0	0	0	0	0
5156	PDGFRA	1	0	0	0	0	0
5159	PDGFRB	0	0	0	0	0	0
5157	PDGFRL	0	0	0	0	0	0
5162	PDHB	0	0	0	0	0	0
9601	PDIA4	0	0	0	0	0	0
5165	PDK3	0	0	0	0	0	0
10630	PDPN	0	0	0	0	0	0
23244	PDS5A	0	0	0	0	1	0
23024	PDZRN3	0	0	0	0	0	0
5175	PECAM1	0	0	0	0	0	0
57161	PELI2	0	0	0	0	1	0
5187	PER1	0	0	0	0	0	0
8863	PER3	0	0	0	0	0	0
5194	PEX13	0	0	0	1	0	0
5202	PFDN2	0	0	0	0	0	0
5203	PFDN4	0	0	0	0	0	0
5209	PFKFB3	0	0	0	0	0	0
5241	PGR	0	0	0	0	0	1
7262	PHLDA2	0	0	0	0	0	0
55022	PID1	0	0	0	0	0	0
63895	PIEZO2	0	0	0	0	0	0
51227	PIGP	0	0	0	0	0	0
113791	PIK3IP1	0	0	0	0	0	0
5311	PKD2	0	0	1	0	0	1
81579	PLA2G12A	0	0	0	0	0	0
5325	PLAGL1	0	0	0	0	1	1
23228	PLCL2	0	0	0	0	0	0
79156	PLEKHF1	0	0	0	0	0	0
57088	PLSCR4	0	0	0	0	0	0
5360	PLTP	0	0	0	0	1	1
83483	PLVAP	0	0	0	1	0	0

57125	PLXDC1	0	0	0	0	0	0
5366	PMAIP1	0	0	0	0	0	0
56902	PNO1	0	0	0	0	0	0
10957	PNRC1	0	0	0	0	0	0
56983	POGLUT1	0	0	0	0	0	0
5425	POLD2	0	0	0	0	0	0
5427	POLE2	0	0	0	0	0	0
9533	POLR1C	0	0	0	0	0	0
5437	POLR2H	0	0	0	0	0	0
5440	POLR2K	0	0	1	0	0	0
8611	PPAP2A	0	0	1	0	0	0
8613	PPAP2B	0	0	0	0	0	0
5471	PPAT	0	0	0	0	0	0
8495	PPFIBP2	0	0	0	0	0	0
9647	PPM1F	0	0	1	0	0	1
23645	PPP1R15A	0	0	0	0	0	0
26051	PPP1R16B	0	0	0	0	0	0
5531	PPP4C	0	0	0	0	0	0
9055	PRC1	0	0	0	0	0	0
5549	PRELP	0	0	0	1	0	0
5571	PRKAG1	0	0	0	0	0	0
112464	PRKCDBP	0	0	0	0	0	0
5583	PRKCH	0	0	0	0	0	0
5587	PRKD1	0	0	0	0	0	0
23683	PRKD3	0	0	0	1	0	1
5627	PROS1	0	0	0	0	0	0
55660	PRPF40A	0	0	0	0	0	0
55851	PSENEN	0	0	0	0	0	0
29893	PSMC3IP	0	0	0	0	0	0
8624	PSMG1	0	0	0	0	0	0
5730	PTGDS	0	0	0	0	0	0
5734	PTGER4	0	0	0	0	0	0
5737	PTGFR	0	0	0	0	0	0
5743	PTGS2	0	0	0	0	0	0
5764	PTN	0	0	0	0	0	0
5789	PTPRD	0	0	0	1	0	0
5797	PTPRM	0	0	0	0	0	0
284119	PTRF	0	0	0	0	0	0
51651	PTRH2	0	0	0	0	0	0
9232	PTTG1	0	0	0	0	0	0
26255	PTTG3P	0	0	0	0	0	0
5813	PURA	0	0	0	0	0	0
54517	PUS7	0	0	0	0	0	0
221749	PXDC1	0	0	0	0	0	0
5827	PXMP2	0	0	0	0	1	0
5860	QDPR	0	0	0	0	0	0
9444	QKI	0	0	0	0	0	0
22841	RAB11FIP2	0	0	0	0	0	0
23682	RAB38	0	0	0	0	0	0
5876	RABGGTB	0	0	0	0	0	0
29127	RACGAP1	0	0	0	0	0	1
5888	RAD51	0	0	0	0	1	0

10635	RAD51AP1	0	0	0	0	0	0
5889	RAD51C	0	0	0	0	0	0
9693	RAPGEF2	0	0	0	0	0	0
5918	RARRES1	0	0	0	0	0	0
5919	RARRES2	0	0	0	0	0	0
54922	RASIP1	0	0	0	0	0	0
51285	RASL12	0	0	0	0	0	0
9770	RASSF2	0	0	0	0	0	0
5931	RBBP7	0	0	0	0	0	0
5932	RBBP8	0	0	0	0	0	0
23543	RBFOX2	0	0	0	0	0	0
64080	RBKS	0	0	0	0	0	0
10181	RBM5	0	0	0	0	0	0
5937	RBMS1	0	0	0	0	0	0
3516	RBPJ	0	0	0	0	0	0
10231	RCAN2	0	0	0	0	0	0
1102	RCBTB2	0	0	0	0	0	0
57333	RCN3	0	0	0	0	0	0
8434	RECK	0	1	0	0	1	0
5649	RELN	0	0	0	0	0	0
5979	RET	0	0	0	0	0	0
23180	RFTN1	0	0	0	0	0	0
28984	RGCC	0	0	0	0	0	0
23179	RGL1	0	0	0	0	0	0
5997	RGS2	0	0	0	0	0	0
9028	RHBDL1	0	0	0	0	0	0
6019	RLN2	0	0	0	0	0	0
55005	RMND1	0	0	0	0	0	0
64795	RMND5A	0	0	0	0	0	0
10535	RNASEH2A	0	0	0	0	0	0
55819	RNF130	0	0	0	1	0	0
9781	RNF144A	0	0	0	0	0	0
6091	ROBO1	0	0	0	0	0	0
4919	ROR1	0	0	0	0	0	1
6119	RPA3	0	0	0	0	1	0
6184	RPN1	0	0	0	0	0	0
10799	RPP40	0	0	0	0	0	0
6196	RPS6KA2	0	0	0	0	0	0
6240	RRM1	0	0	0	0	0	0
6241	RRM2	0	0	0	0	0	0
23212	RRS1	0	0	0	0	0	0
862	RUNX1T1	0	0	0	1	0	0
8607	RUVBL1	0	0	0	0	0	0
6281	S100A10	0	0	0	0	0	0
57402	S100A14	0	0	0	0	0	0
6275	S100A4	0	0	0	0	0	0
1901	S1PR1	0	0	0	0	0	0
29901	SAC3D1	0	0	0	0	0	0
23328	SASH1	0	0	0	0	0	0
60485	SAV1	0	0	0	0	0	0
51435	SCARA3	0	0	0	0	0	0
23541	SEC14L2	0	0	0	0	0	0

10483	SEC23B	0	0	0	0	0	0
6403	SELP	1	0	0	1	0	0
9037	SEMA5A	0	0	0	0	0	0
55752	SEPT11	0	0	0	0	0	0
27230	SERP1	0	0	1	0	0	0
12	SERPINA3	0	0	0	0	0	0
5176	SERPINF1	0	0	0	0	0	0
710	SERPING1	0	0	0	0	1	0
6421	SFPQ	0	0	0	0	0	0
6422	SFRP1	0	0	0	0	0	0
6424	SFRP4	0	0	0	0	0	0
6446	SGK1	0	0	0	0	0	0
9467	SH3BP5	0	0	0	0	0	0
79801	SHCBP1	0	0	0	0	0	0
6474	SHOX2	0	0	0	1	0	0
357	SHROOM2	0	0	0	0	0	0
6478	SIAH2	0	0	0	0	0	0
140885	SIRPA	0	0	0	0	0	0
8935	SKAP2	0	0	0	0	0	0
51296	SLC15A3	0	0	0	0	0	0
9120	SLC16A6	0	0	0	0	0	0
10560	SLC19A2	0	0	0	0	0	1
10166	SLC25A15	0	0	0	0	1	0
10478	SLC25A17	0	0	0	0	0	0
29957	SLC25A24	0	0	0	0	0	0
11001	SLC27A2	0	0	0	0	0	0
6513	SLC2A1	0	0	0	0	0	0
25800	SLC39A6	0	0	0	0	0	0
29015	SLC43A3	0	0	0	0	1	0
9497	SLC4A7	0	0	0	0	0	0
6541	SLC7A1	0	0	0	0	0	0
9368	SLC9A3R1	0	0	0	0	0	0
9353	SLIT2	0	0	0	0	0	0
6586	SLIT3	0	0	0	0	0	0
6599	SMARCC1	0	0	0	0	0	0
10592	SMC2	0	0	0	0	0	0
6591	SNAI2	0	0	0	0	0	0
9627	SNCAIP	1	0	0	1	0	0
54861	SNRK	0	0	1	1	0	1
6632	SNRPD1	0	0	0	0	0	0
6634	SNRPD3	0	0	0	0	0	1
6645	SNTB2	0	0	0	0	0	0
28966	SNX24	0	0	0	0	0	0
55084	SOBP	0	0	0	0	0	0
8835	SOCS2	0	0	0	0	0	0
9655	SOCS5	0	0	0	1	0	0
53340	SPA17	0	0	0	0	0	0
10615	SPAG5	0	0	0	0	0	0
6678	SPARC	0	0	0	0	0	0
8404	SPARCL1	0	0	0	0	0	0
26010	SPATS2L	0	0	0	0	0	0
27290	SPINK4	0	0	0	0	0	0

10653	SPINT2	0	0	0	0	0	1
10418	SPON1	0	0	0	0	0	0
10417	SPON2	0	0	0	0	0	0
10252	SPRY1	0	0	0	0	0	0
10253	SPRY2	0	0	0	0	1	0
6728	SRP19	0	0	0	0	0	0
6732	SRPK1	0	0	0	0	0	0
6733	SRPK2	0	0	0	0	0	0
8406	SRPX	0	0	0	0	0	1
6426	SRSF1	0	0	0	0	0	0
6429	SRSF4	0	0	0	0	0	0
54434	SSH1	0	0	0	0	0	0
8082	SSPN	0	0	0	0	0	0
6747	SSR3	0	0	0	0	0	0
6764	ST5	0	0	0	0	0	0
6480	ST6GAL1	0	0	0	0	0	0
23166	STAB1	0	0	0	0	0	0
10617	STAMBP	0	0	0	0	0	0
6776	STAT5A	0	0	0	0	0	0
6777	STAT5B	0	0	0	0	0	0
6781	STC1	0	0	0	0	0	0
8614	STC2	0	0	0	0	0	0
3925	STMN1	0	0	0	0	0	0
30968	STOML2	0	0	0	0	0	0
11037	STON1	0	0	0	0	0	0
11171	STRAP	0	0	0	0	0	0
6812	STXBP1	0	0	0	0	0	0
10923	SUB1	0	0	0	0	0	0
23512	SUZ12	0	0	0	0	0	0
6840	SVIL	0	0	0	0	0	0
85360	SYDE1	0	0	0	0	0	0
79953	SYNDIG1	0	0	0	0	0	0
23345	SYNE1	0	0	0	0	0	0
11346	SYNPO	0	0	1	0	1	1
6867	TACC1	0	1	0	0	0	0
10460	TACC3	0	0	0	0	0	0
6876	TAGLN	0	0	0	0	0	0
6895	TARBP2	0	0	0	0	0	0
54662	TBC1D13	0	0	0	0	0	0
23102	TBC1D2B	0	0	0	0	0	1
9779	TBC1D5	0	0	0	0	0	0
6905	TBCE	0	0	0	0	0	0
6925	TCF4	0	0	1	0	0	1
83439	TCF7L1	0	0	0	0	1	0
6934	TCF7L2	0	0	0	0	0	0
23371	TENC1	0	0	0	0	0	0
7942	TFEB	0	0	0	0	0	0
7031	TFF1	0	0	0	0	0	0
7033	TFF3	0	0	0	0	0	0
7037	TFRC	0	0	0	0	0	0
7041	TGFB1I1	0	0	0	0	0	0
7043	TGFB3	0	0	0	0	0	0

7048	TGFBR2	0	1	0	0	1	0
7056	THBD	0	0	0	0	0	0
7059	THBS3	0	0	0	0	0	0
221981	THSD7A	0	0	0	0	0	1
8914	TIMELESS	0	0	0	0	0	0
10440	TIMM17A	0	0	0	0	1	0
10245	TIMM17B	0	0	0	0	0	0
100287932	TIMM23	0	0	0	0	0	0
1678	TIMM8A	0	0	0	0	0	0
54962	TIPIN	0	0	0	0	0	0
7083	TK1	0	0	0	0	1	1
79838	TMC5	0	0	0	0	0	0
55281	TMEM140	0	0	0	0	0	0
28978	TMEM14A	1	0	0	1	0	1
80775	TMEM177	0	0	0	0	0	0
79188	TMEM43	0	0	0	0	0	0
55706	TMEM48	0	0	0	0	0	0
83460	TMEM93	0	0	0	0	0	0
27346	TMEM97	0	0	0	0	0	1
7112	TMPO	0	0	0	0	0	0
8600	TNFSF11	0	0	0	0	0	0
8742	TNFSF12	0	0	0	0	0	0
7138	TNNT1	0	0	0	0	0	0
7145	TNS1	0	0	1	1	0	1
100188893	TOMM6	0	0	0	0	0	0
9868	TOMM70A	0	0	0	0	0	0
7153	TOP2A	0	0	0	0	0	0
7162	TPBG	0	0	0	0	0	0
51002	TPRKB	0	0	0	1	0	0
8460	TPST1	0	0	0	0	0	0
9697	TRAM2	0	0	0	0	0	0
27095	TRAPPC3	0	0	0	0	0	0
23321	TRIM2	0	0	0	0	0	0
55128	TRIM68	0	0	0	0	0	0
11078	TRIOBP	0	0	0	0	0	0
9322	TRIP10	0	0	0	0	0	0
9319	TRIP13	0	0	0	0	0	0
55039	TRMT12	0	0	0	0	0	0
7220	TRPC1	0	0	0	0	0	0
7227	TRPS1	0	0	0	0	1	0
8848	TSC22D1	0	0	0	0	0	0
10102	TSFM	0	0	0	0	0	0
27075	TSPAN13	0	0	0	0	0	0
7102	TSPAN7	0	0	0	0	1	0
64061	TSPYL2	0	0	0	0	0	0
55720	TSR1	0	0	0	0	0	0
7260	TSSC1	0	0	0	1	0	0
64927	TTC23	0	0	0	0	0	0
23331	TTC28	0	0	0	0	0	0
22996	TTC39A	0	0	0	0	0	0
7272	TTK	0	0	0	0	0	0
23170	TTLL12	0	0	0	0	0	0

7283	TUBG1	0	0	0	0	0	0
7291	TWIST1	0	0	0	0	0	0
7295	TXN	0	0	0	0	0	0
10628	TXNIP	0	0	0	0	0	0
7298	TYMS	0	0	0	0	0	0
11065	UBE2C	0	0	0	0	0	0
7323	UBE2D3	0	0	0	0	0	0
7336	UBE2V2	0	0	0	0	0	0
56061	UBFD1	0	0	0	0	0	0
7371	UCK2	0	0	0	0	0	0
56886	UGGT1	0	0	0	0	0	0
7372	UMPS	0	0	0	0	0	0
7374	UNG	0	0	0	0	0	0
27089	UQCRQ	0	0	0	0	0	0
9816	URB2	0	0	0	0	0	0
9097	USP14	0	0	0	0	0	0
10713	USP39	0	0	0	0	0	0
7874	USP7	0	0	0	0	0	0
10813	UTP14A	0	0	0	0	0	0
7405	UVRAG	0	0	0	0	0	0
10493	VAT1	0	0	0	0	0	0
7419	VDAC3	0	0	0	0	0	0
7424	VEGFC	0	0	0	0	0	0
7431	VIM	0	0	0	0	0	0
7443	VRK1	0	0	0	0	0	0
7444	VRK2	0	0	0	0	0	0
196740	VSTM4	0	0	0	0	0	0
7450	VWF	0	0	0	0	0	0
11169	WDHD1	0	0	0	0	0	0
55759	WDR12	0	0	0	0	0	0
10885	WDR3	0	0	0	0	0	0
55093	WDYHV1	0	0	0	0	0	0
7456	WIPF1	0	0	0	0	0	0
8839	WISP2	0	0	0	0	0	0
25937	WWTR1	0	0	0	0	0	0
51067	YARS2	0	0	0	0	0	0
10730	YME1L1	0	0	0	0	1	0
7704	ZBTB16	0	0	0	0	0	0
26137	ZBTB20	0	0	0	0	0	0
23174	ZCCHC14	0	0	0	0	0	0
219654	ZCCHC24	1	0	1	1	0	1
6935	ZEB1	0	0	0	0	0	0
9839	ZEB2	1	0	0	0	0	0
79776	ZFHX4	1	1	1	1	1	1
64397	ZFP106	0	0	0	1	0	0
7538	ZFP36	0	0	0	0	0	0
677	ZFP36L1	0	0	0	0	0	0
678	ZFP36L2	0	0	0	0	0	0
23414	ZFPM2	0	0	0	0	0	0
64393	ZMAT3	0	0	0	0	0	0
7767	ZNF224	0	0	0	0	0	0
54816	ZNF280D	0	0	0	0	0	0

55422	ZNF331	0	0	0	0	0	0
23090	ZNF423	0	0	0	0	0	0
79818	ZNF552	0	0	0	0	1	0
84914	ZNF587	0	0	0	0	0	0
100293516	ZNF587B	0	0	0	0	0	0
11130	ZWINT	0	0	0	0	1	0
7791	ZYX	0	0	0	1	1	0
